# T1 Mapping as an indication of diffuse, diabetes-related myocardial collagen deposition

**DOI:** 10.1186/1532-429X-15-S1-P171

**Published:** 2013-01-30

**Authors:** Anna Schmidt, Kelvin Chow, David Lau, Richard B Thompson, Matthias G Friedrich

**Affiliations:** 1 Cardiovascular Sciences, University of Calgary, Calgary, AB, Canada; 2 Stephenson Cardiac MR Centre, University of Calgary, Calgary, AB, Canada; 3 University of Alberta, Edmonton, AB, Canada; 4 Montreal Heart Institute, Montreal, QC, Canada

## Background

Type 2 diabetes (T2D) is an independent risk factor for adverse cardiac events, such as hypertension and myocardial infarction. The underlying biochemical changes associated with diabetes have been shown to increase collagen deposition and cross-linking, which may lead to microscopic alterations in the myocardial structure that precede measurable changes in cardiac function. Non-invasive imaging methods are important for risk stratification of asymptomatic patients and to gain a better understanding of the pathophysiology of diabetic heart disease. T1 mapping is a novel Cardiovascular Magnetic Resonance Imaging (CMR) technique for quantification of increased extracellular volume fraction that occurs with diffuse collagen replacement. The objective of this research is to examine post-contrast myocardial T1 values in asymptomatic T2D patients.

## Methods

**Figure 1:**
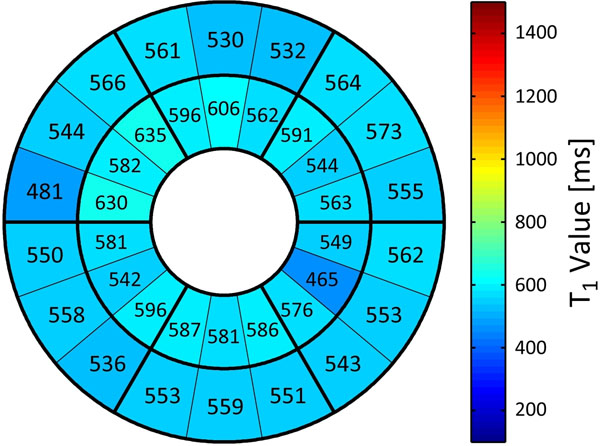
Myocardial segment plot of post-contrast myocardial T1 values in a diabetes patient, showing basal and mid slice with an overall average T1 value of 562±33 ms.

Patients diagnosed with type 2 diabetes (HbA1c 7.5-9.9%; mean age 53±8; n=10), and healthy, non-diabetic age-matched controls (mean age 47±10; n=10) were assessed at the Stephenson CMR Centre in Calgary using a clinical 1.5 T scanner. Medical history and ECG were reviewed to rule out ischemic heart disease. Eligible patients underwent a CMR scan including quantitative T1 mapping (SASHA) before and 15 minutes after 0.15 mmol/kg of Gd-DTPA (Magnevist) contrast injection, with subsequent evaluation of mid and basal slices for segment and global T1 values (Figure 1). Additionally, standard CMR protocols were used to assess function, and Late Gadolinium Enhancement (LGE).

## Results

A significant difference in post-contrast myocardial T1 values was observed in patients as compared to controls (580 ± 54 and 623 ± 31 ms; p < 0.05). No significant differences in functional parameters were found between patients and controls; both had normal systolic function (LV ejection fraction 57.9 ± 2.6 and 57.8 ± 2.9%, respectively), although T2D patients had a slightly reduced indexed LV end diastolic volumes. No regional areas of LGE were detected in either group.

## Conclusions

The observed decrease in T1 mapping times reflect an increased extracellular volume fraction, and may reflect globally increased collagen deposition in diabetic myocardium. This finding compliments previous findings by this group of preclinical changes in microvascular function in diabetes. Further studies are required to assess the pathophysiologic context and prognostic impact.

## Funding

none

